# Non-functioning pituitary macroadenoma following surgery: long-term outcomes and development of an optimal follow-up strategy

**DOI:** 10.3389/fsurg.2023.1129387

**Published:** 2023-07-12

**Authors:** Ziad Hussein, Joan Grieve, Neil Dorward, Katherine Miszkiel, Michael Kosmin, Naomi Fersht, Pierre Marc Bouloux, Zane Jaunmuktane, Stephanie E. Baldeweg, Hani J. Marcus

**Affiliations:** ^1^ Department of Diabetes and Endocrinology, Sheffield Teaching Hospitals NHS Foundation Trust, Sheffield, United Kingdom; ^2^ Department of Diabetes and Endocrinology, University College London Hospital NHS Foundation Trust, London, United Kingdom; ^3^ Department of Neurosurgery, National Hospital for Neurology and Neurosurgery, London, United Kingdom; ^4^ Department of Clinical Oncology, University College London Hospitals, London, United Kingdom; ^5^ Centre for Neuroendocrinology, Royal Free Campus, University College Medical School, University College London, London, United Kingdom; ^6^ Institute of Neurology, University College London, London, United Kingdom; ^7^ Division of Medicine, Department of Experimental and Translational Medicine, Centre for Obesity and Metabolism, University College London, London, United Kingdom

**Keywords:** non functioning pituitary adenoma, radiotherapy, transsphenoidal surgery, recurrence, follow-up strategy

## Abstract

**Objectives:**

Recurrence and regrowth of non-functioning pituitary macroadenomas (NFPMs) after surgery are common but remain unpredictable. Therefore, the optimal timing and frequency of follow-up imaging remain to be determined. We sought to determine the long-term surgical outcomes of NFPMs following surgery and develop an optimal follow-up strategy.

**Methods:**

Patients underwent surgery for NFPMs between 1987 and 2018, with a follow-up of 6 months or more, were identified. Demographics, presentation, management, histology, imaging, and surgical outcomes were retrospectively collected.

**Results:**

In total, 383 patients were included; 256 were men (256/383; 67%) with median follow-up of 8 years. Following primary surgery, 229 patients (229/383; 60%) achieved complete resection. Of those, 28 (28/229; 11%) developed recurrence, including six needed secondary surgery (6/229; 3%). The rate of complete resection improved over time; in the last quartile of cases, 77 achieved complete resection (77/95; 81%). Reoperation-free survival at 5, 10 and 15 years was 99%, 94% and 94%, respectively. NFPMs were incompletely resected in 154 patients (154/383; 40%); of those, 106 (106/154; 69%) had regrowth, and 84 (84/154; 55%) required reoperation. Surgical reintervention-free survival at 5, 10 and 15 years was 74%,49% and 35%, respectively. Young age and cavernous sinus invasion were risk factors for undergoing reoperation (*P* < 0.001 and *P* < 0.0001, respectively) and radiotherapy (*P* = 0.003 and *P* < 0.001, respectively). Patients with residual tumour required reoperation earlier than those underwent complete resection (*P* = 0.02). Radiotherapy to control tumour regrowth was delivered to 65 patients (65/383; 17%) after median time of 1 year following surgery. Radiotherapy was administered more in patients with regrowth of residual disease (61/106; 58%) than those who had NFPMs recurrence (4/28; 14%) (*P* ≤ 0.001) Following postoperative radiotherapy, one patient (1/65; 2%) had evidence of regrowth, seven (7/65; 11%) had tumour regression on imaging, and no patients underwent further surgery.

**Conclusions:**

NFPMs recurrence and regrowth are common, particularly in patients with residual disease post-operatively. We propose a follow-up strategy based on stratifying patients as “low risk” if there is no residual tumour, with increasing scan intervals, or “high risk” if there is a residual tumour, with annual scans for at least five years and extended lifelong surveillance after that.

## Introduction

Adenohypophyseal tumours are well-differentiated pituitary neuroendocrine tumours (PitNETs) of the pituitary gland (1) expressing distinct morphologic, molecular, and clinical differences. Non-secretory gonadotropin-expressing subtype comprises around 80–90% of PitNETs (2–4) and varies in clinical presentation from an incidental finding on imaging to large macroadenomas damaging the pituitary gland and the optic pathway (5, 6). Consequently, hypopituitarism, visual compromise and headache are common clinical manifestations (7).

Transsphenoidal surgery is the gold standard therapy for pituitary macroadenomas compressing the optic apparatus. Recurrence and tumour progression following operation is challenging and can be managed with further surgical resection or adjuvant radiotherapy for patients with inoperable tumours or relatively large residual disease (8, 9). To date, there is no effective medical therapy for non-functioning pituitary adenomas (10).

There are no clear predictive clinical or histological factors for non-functioning pituitary macroadenomas (NFPMs) recurrence following surgery (11). Therefore, postoperative neuroradiological surveillance is variable, and imaging frequency is often debated. Developing risk stratification of patients is required to apply the optimal treatment modality.

This study aimed to investigate surgical outcomes in patients with NFPMs. The primary aim is to assess the natural course of tumour recurrence and regrowth following surgical intervention and propose an optimal neuroimaging follow-up strategy.

## Methods

Ethics approval to conduct this study was obtained from Westminster Research Ethics Committee on 07/04/2020. The Strengthening the Reporting of Observational Studies in Epidemiology (STROBE) Statement was used to prepare this section of the manuscript (12). This study was a single-centre cohort study including all patients who underwent surgical resection for NFPMs between 1987 and 2018 with or without radiotherapy and had a follow-up duration of more than six months. The study was conducted at the National Hospital for Neurology and Neurosurgery based in London. Surgical resection was performed mainly by three experienced neurosurgeons: MP and JG, who adopted a microscopic transsphenoidal approach, and ND, who adopted an endoscopic transsphenoidal approach. A retrospective review of medical case notes was performed.

## Data collection

The diagnosis of NFPMs was based on the absence of clinical and biochemical evidence of functioning tumours. Data were collected on patients' demographics, clinical presentation, pre-operative imaging, treatment modalities, and postoperative recurrence and regrowth. Pre-operative imaging included craniocaudal, transverse, and anteroposterior dimensions, and tumour volume was calculated using the ellipsoid formula [43π (a.b.c)] (13).

**Figure 1:**
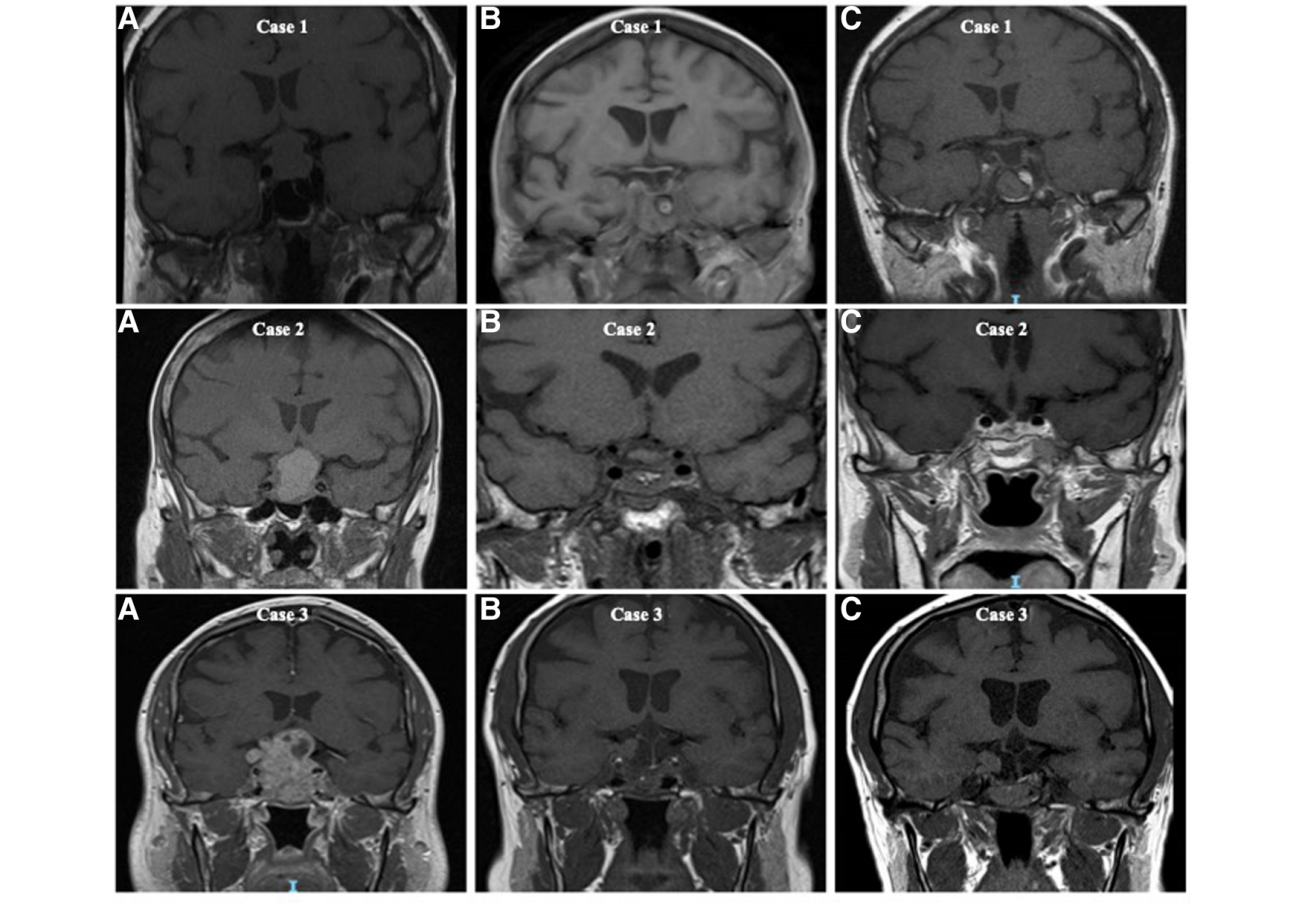
T1-weighted coronal magnetic resonance imaging (MRI) illustrating the extent of surgical resection of non-functioning pituitary macroadenomas and the classification of complete resection, residual disease and sellar tissue of uncertain significance based on the appearance on postoperative imaging. Case 1 (**A**) preoperative MRI, (**B**) initial postoperative MRI with complete excision reported, and (**C**) 5 years following surgery. Case 2 (**A**) preoperative MRI, (**B**) initial postoperative MRI with residual sellar tissue of uncertain significance reported, and (**C**) 5 years following surgery. Case 3 (**A**) preoperative MRI, (**B**) initial postoperative MRI with obvious residual disease reported, and (**C**) 5 years post surgery.

The initial decision to operate was made following a discussion in the pituitary multidisciplinary meeting. Primary surgery was performed for patients with NFPMs threatening or compressing the optic pathway or when there was diagnostic uncertainty. In all cases, tumour specimens underwent immunohistological analysis to identify the type of pituitary adenoma. MIB-1 monoclonal antibody was used to detect Ki-67 antigen in formalin-fixed, paraffin-embedded tissues. Ki-67 expression of more than 3% was considered high (14, 15). All patients had post-operative Magnetic Resonance Imaging (MRI) or, if this was contra-indicated, Computed Tomography (CT) between 3 and 6 months post-surgery to assess the extent of surgical resection. All imagings were reported by independent neuroradiologists as either complete resection, incomplete resection, or residual sellar tissue of uncertain significance (Figure 1). Tumour recurrence was considered when there was tumour reappearance on follow-up imaging after total resection, while regrowth was considered when there was a growth of the residual lesion post subtotal resection, as reported by an independent neuroradiologist. The decision to offer reoperation and administer radiotherapy was based on the assessment of the patient's vision, their associated imaging findings, and discussion in the pituitary multidisciplinary meeting.

## Statistical analysis

For the purposes of statistical analysis, residual sellar tissue of uncertain significance was grouped with complete resection, indicating that a case was considered as a complete resection unless an obvious residual disease had been present. This decision was made post-hoc to facilitate comparison with other studies.

Primary data were evaluated using descriptive statistics. Mean and standard deviation (SD) were used to describe continuous variables. Median and interquartile range (IQR) were used to describe data not normally distributed. The chi-square test was used to compare categorical variables. The Pearson correlation test was used to assess the correlation between nominal and numerical variables. Recurrence-free curves were generated by the Kaplan–Meier method, and the evaluations of the differences in the various sub-groups were done by the log-rank test. The Spearman analysis test was used to measure the correlation between continuous and nominal variables. A *P* value <0.05 was considered statistically significant. IBM SPSS statistics version 28 was used in this study.

## Results

### Patients' characteristics, clinical and radiological features at presentation

In total, 383 patients were identified for this study; 256 (256/383; 67%) were men, and 127 (127/383; 33%) were women with a median follow-up duration of 8 years (IQR 5–10). The median age at presentation for the full cohort was 57 years (IQR 48–67).

The leading presenting manifestation was visual impairment (228/383; 60%). On formal ophthalmic assessment, 262 patients (262/383; 68%) had signs of visual field defect caused by optic compression. Hypopituitarism at presentation occurred in 58 patients (58/377, 15%); however, on preoperative endocrine screening (available for 377 patients), 235 patients (235/377; 62%) had evidence of at least one pituitary hormone deficiency. Sixty-six patients had their pituitary macroadenoma detected incidentally on radiological imaging (66/383; 17%) and due to headache in 41 patients (41/383; 11%). Twelve patients (12/377; 3%) were admitted with pituitary apoplexy.

With respect to radiological findings before surgery, data were available for 379 patients (Table 1). Fourteen patients (14/379; 4%) had a macroadenoma confined to the intrasellar space, 357 patients (357/379; 94%) had NFPMs abutting or compressing the optic apparatus, and 124 patients (124/379; 33%) had evidence of cavernous and sphenoid sinuses invasion (Table 1). NFPMs dimensions and volume on preoperative imaging were available for 317 patients; the correlation between tumour size with tumour recurrence and regrowth is shown in Table 2. Data of those with cavernous and sphenoid sinuses invasion were analysed separately and compared with the rest of the cohort (Table 3).

**Table 1:** Radiological features of non-functioning pituitary macroadenomas at presentation. Imaging data at time of diagnosis were available for 379 patients.

Macroadenomas with/without suprasellar extension but not touching the optic chiasm	14 (14/379; 4%)
Macroadenoma abutting or compressing the optic apparatus	357 (357/379; 94%)
Cavernous Sinus invasion	115 (115/379; 30%)
Sphenoid invasion	21 (21/379; 6%)
Clivus invasion	9 (9/379; 2%)
Extension to lateral ventricle	5 (5/379; 1%)
Nasopharynx invasion	2 (2/379; <1%)
Temporal lobe invasion	1 (1/379; <1%)
Knosp classification of pituitary macroadenomas	
Grade I	143/379 (38%)
Grade II	126/379 (33%)
Grade III A	63/379 (17%)
Grade III B	8/379 (2%)
Grade IV	43/379 (11%)

**Table 2:** The incidence of non-functioning pituitary macroadenoma recurrence and regrowth following primary surgery based on patient's age, gender, and technique of surgical visualization for the full cohort.

	Tumour recurrence/ regrowth	No tumour recurrence/ regrowth	*P* value
Median age (IQR)	54 years (44–61)	59 years (49–68)	0.001
Gender			
Male	81/256 (32%)	175/256 (68%)	0.3
Female	47/127 (37%)	80/127 (63%)	
Tumour dimensions and volume on preoperative imaging			
Craniocaudal diameter (IQR)	3.2 cm (2.4–3.9)	2.5 cm (1.9–3.2)	0.001
Transverse diameter (IQR)	2.7 cm (2.1–3)	2.2 cm (1.8–2.7)	0.001
Anteroposterior diameter (IQR)	2.3 cm (1.9–2.6)	1.9 cm (1.6–2.3)	0.001
Volume (IQR)	10.5 cm^3^ (5–16)	5.8 cm^3^ (3–10)	0.001
Surgical technique			
Microscopic surgery	103/316 (33%)	213/316 (67%)	0.2
Endoscopic surgery	10/44 (22%)	34/44 (78%)	

Cm, centimetre; IQR, interquartile range.

**Table 3:** Data of patients with cavernous and sphenoid sinuses invasion and those without on imaging at time of non-functioning pituitary macroadenoma diagnosis. Imaging data at time of diagnosis were available for 379 patients. Combined data for Ki-67 and preoperative radiology were reported in 374 patients. Data of preoperative imaging for patients who underwent secondary surgery were recorded for 87 patients.

	Cavernous and sphenoid sinuses invasion	No invasion	*P* value
Sex (Male)	83/124 (67%)	170/255 (67%)	1
Age	59 years (48–67)	57 (46–66)	0.06
Median tumour volume (IQR) on preoperative imaging	10 cm^3^ (6–17)	5 cm^3^ (3–10)	0.001
Patients needed secondary surgery	34/124 (32%)	53/255 (21%)	0.2
Median timing (IQR) of surgical reintervention	4.5 years (2–11)	7 years (5–10)	0.3
Ki-67 > 3%	15/120 (13%)	28/254 (11%)	0.6
Patients received radiotherapy	40/124 (32%)	25/255 (10%)	<0.001

Cm, centimetre; IQR, interquartile range.

### Surgical management of NFPMs

For primary surgery, most patients underwent transsphenoidal resection of NFPMs (378/383; 99%), and only five patients had transcranial surgery (5/383; 1%) (*P* < 0.001). Most of the patients in this cohort (360/383; 94%) underwent their primary surgery in our centre, while 23 patients (23/383; 6%) had their primary operation in other hospitals but were later referred to our hospital for ongoing treatment for recurrence or regrowth.

Following primary surgery, a total of 13 patients (13/383; 4%) had immediate surgical complications requiring intervention. These complications included eight patients with CSF leak (8/383; 2%), three patients with sellar haemorrhage (3/383; 1%), one patient with carotid injury (1/383; 0.3%), and one patient with an abdominal graft site haematoma (1/383; 0.3%). There were no postoperative deaths within 30 days of primary surgery in the cohort.

**Figure 2:**
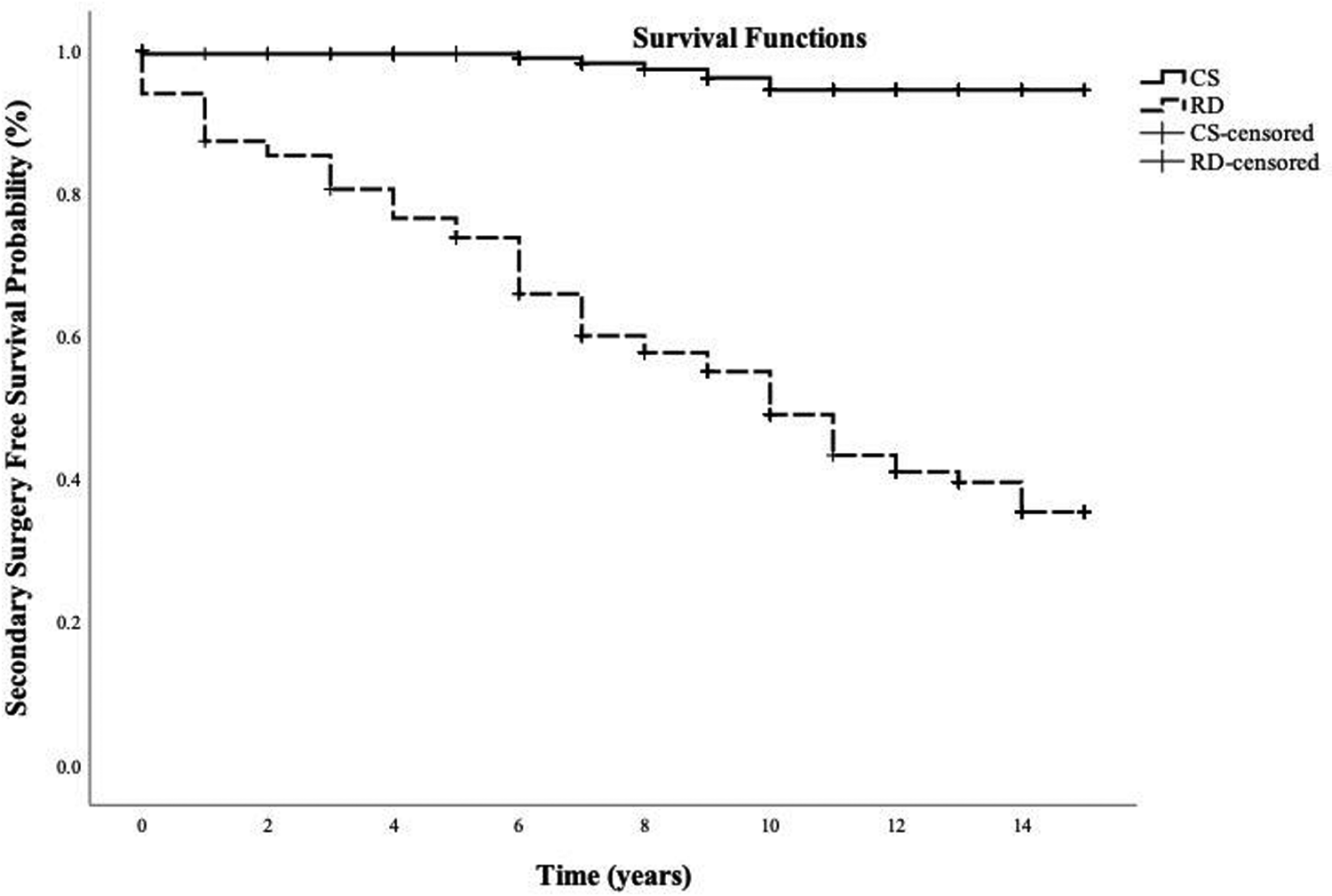
Kaplan-Meier curve illustrating surgical reintervention-free survival probability for patients with complete resection of non-functioning pituitary macroadenomas and those with residual disease following primary surgery. Follow-up time was measured in years from the initial surgery. CS, complete resection; RD, residual disease.

Total resection was achieved in 229 patients (229/383; 60%). The rate of complete resection improved over time; in the last quartile of cases, 77 patients achieved complete resection (77/95; 81%). Of those that achieved complete resection, 28 patients (28/229; 11%) had tumour recurrence on imaging after a median follow-up of 3 years (IQR 2–5). Six of these patients (6/229; 3%) had tumour recurrence requiring a second surgery after a median duration of 7 years (IQR 6–10) after their first surgery. Re-operation was via the transsphenoidal approach in all cases. Surgical reintervention-free survival probability for those who had complete resection at 5, 10 and 15 years was 99%, 94% and 94%, respectively (Figure 2).

Residual disease was evident in 154 patients (154/383; 40%) on the first postoperative imaging; of those, 106 (106/154; 69%) had tumour regrowth on imaging after a median follow-up of 3 years (IQR 1–5) and 84 patients (84/154; 55%) needed a second surgery after a median duration of 6 years (IQR 2–9) of their first surgery. Transsphenoidal resection for adenoma recurrence or regrowth was performed in 80 patients (80/84; 95%), while only four patients had transcranial operation (4/84; 5%). Surgical reintervention-free survival probability for patients who underwent incomplete resection of NFPM at 5, 10, and 15 years was 74%, 49% and 35%, respectively (Figure 2).

Patients with residual disease following primary surgery had a higher risk of tumour regrowth than those who underwent complete resection with an Odds ratio of 16 (95% CI = 10–28) (*P* = <0.001). Younger patients were more likely to undergo secondary surgery with a median age of 53 years (IQR 44–61) vs. those patients who didn’t need surgical reintervention at 59 years (IQR 49–68) (*P* = <0.001). Cavernous and sphenoid sinuses invasion on the first postoperative imaging didn't influence the need for further surgical intervention in those with and in those without features of invasion (0.2).

For the entire cohort, larger tumours, as demonstrated on pre-operative imaging, were associated with an increased risk of recurrence and regrowth (Table 2). Younger patients had a higher risk of disease recurrence (*P* ≤ 0.001). Patient's sex and choice of micro-or endoscopic surgical approach were not shown to be risk factors for tumour recurrence and regrowth (Table 2).

### Histological data

Based on hormone expression using immunohistological analysis of the resected tumours, Gonadotroph-expressing NFPMs occurred in 371 patients, while 12 patients had pleurihormonal pituitary adenomas. The extent of Ki-67 expression in the resected pituitary adenomas is shown in Table 4. It was higher in those who required a secondary surgery, but this was not statistically significant; 23% (77/335) of patients with Ki-67 < 3% required re-operation, compared to 30% (13/43) of patients with Ki-67% > 3% (*P* = 0.3). Nor was Ki-67 associated with the timing of the second surgery (*P* = 0.2). However, Ki-67 was higher in those that received postoperative radiotherapy than in those treated with surgery alone (*P* ≤ 0.001) (Table 4).

**Table 4:** Expression of Ki-67, represented in percentage, in the resected pituitary adenomas according to patients’ age, visual deficit, extrasellar extension and treatment modality. Data on Ki-67 expression in the resected adenomas were available in 378 patients. Combined details of Cavernous and sphenoid sinuses invasion on preoperative radiology and Ki-67 were reported in 374 patients.

Ki-67 expression in the resected pituitary adenomas			
Less than 1%	84/378 (22%)		
1–3%	251/378 (66%)		
3–5%	37/378 (10%)		
>5%	6/378 (2%)		
Ki-67	<3%	>3%	*P* value
Median age (IQR)	59 years (48–67)	53 years (45–60)	0.01
Visual loss	225/335 (67%)	32/43 (74%)	0.1
Cavernous and sphenoid sinuses invasion on first imaging	105/331 (32%)	15/43 (35%)	0.9
Patients underwent primary surgery only	258/335 (77%)	30/43 (70%)	0.3
Patients received reoperation for tumour recurrence/regrowth	77/335 (23%)	13/43 (30%)	
Patient treated with adjuvant radiotherapy	49/335 (14%)	16/43 (37%)	0.001

There was a significant inverse relationship between high Ki-67 and age (*P* = 0.01) (Table 4). Ki-67 expression was similar in those with preoperative visual loss and those with normal vision (*P* = 0.1). In addition, high ki-67 more than 3% was not associated with cavernous sinus invasion on preoperative imaging (13/111; 14% with cavernous sinus involvement vs. 30/263; 11% in those without) (*P* = 0.9).

### Radiotherapy

Pituitary radiotherapy to control tumour regrowth was delivered to 65 patients (65/383; 17%) after a median time of 1 year (IQR 1–3) following surgery. Radiotherapy was used more to control tumour progression in those who had regrowth of their residual disease (61/106; 58%) than those patients who had NFPMs recurrence after complete resection (4/28; 14%) (*P* ≤ 0.001). External beam radiotherapy was administered in 63 patients (50.4 Gray in 28 daily fractions over 5.5 weeks), and single-fraction Gamma knife radiosurgery was performed in two patients. One patient (1/65; 2%) showed evidence of tumour regrowth after 23 years from receiving radiotherapy, and seven patients (7/65; 11%) had NFPMs regression on imaging after radiotherapy. None of those treated with irradiation needed further surgery. Four patients (4/65) had a complete resection.

**Figure 3:**
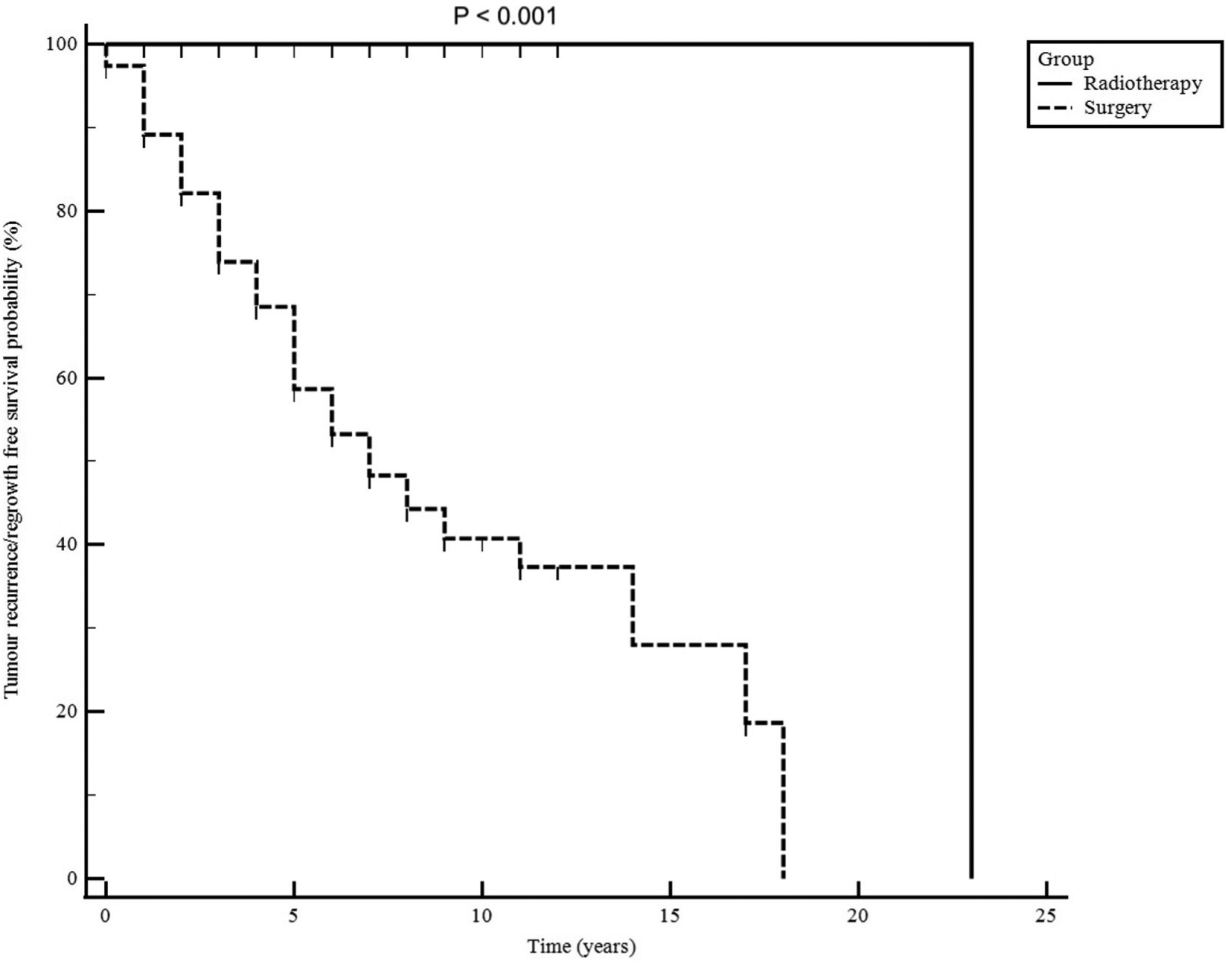
Non-functioning pituitary macroadenomas radiological progression-free survival for patients treated with surgery alone and those treated with postoperative radiotherapy. Follow-up time is shown in years from the first surgery.

Tumour progression-free survival probability after irradiation was 100% at 5, 10 and 15 years (Figure 3). Postoperative radiotherapy was used less in older patients to control residual tumour (*P* = 0.003) and was delivered more often in patients with cavernous and sphenoid sinuses invasion (39/124; 32%) on postoperative imaging than those without (26/255; 10%) (<0.001).

### Visual outcome

Normal visual assessment was reported in 110 patients (110/383; 29%), while 263 patients (263/383; 69%) had evidence of visual impairment before surgery. Nine patients (9/383; 2%) with underlying eye disease were excluded. At the latest review, 81 patients with preoperative visual deficit (81/263; 31%) had normal vision, 155 patients (155/263; 59%) had visual improvement, 18 patients (18/263; 7%) retained their visual impairment, and one patient (1/263; 0.4%) suffered permanent deterioration following surgery. There were no reported cases of radiotherapy-induced optic neuropathy.

## Discussion

### Principal findings

**Figure 4:**
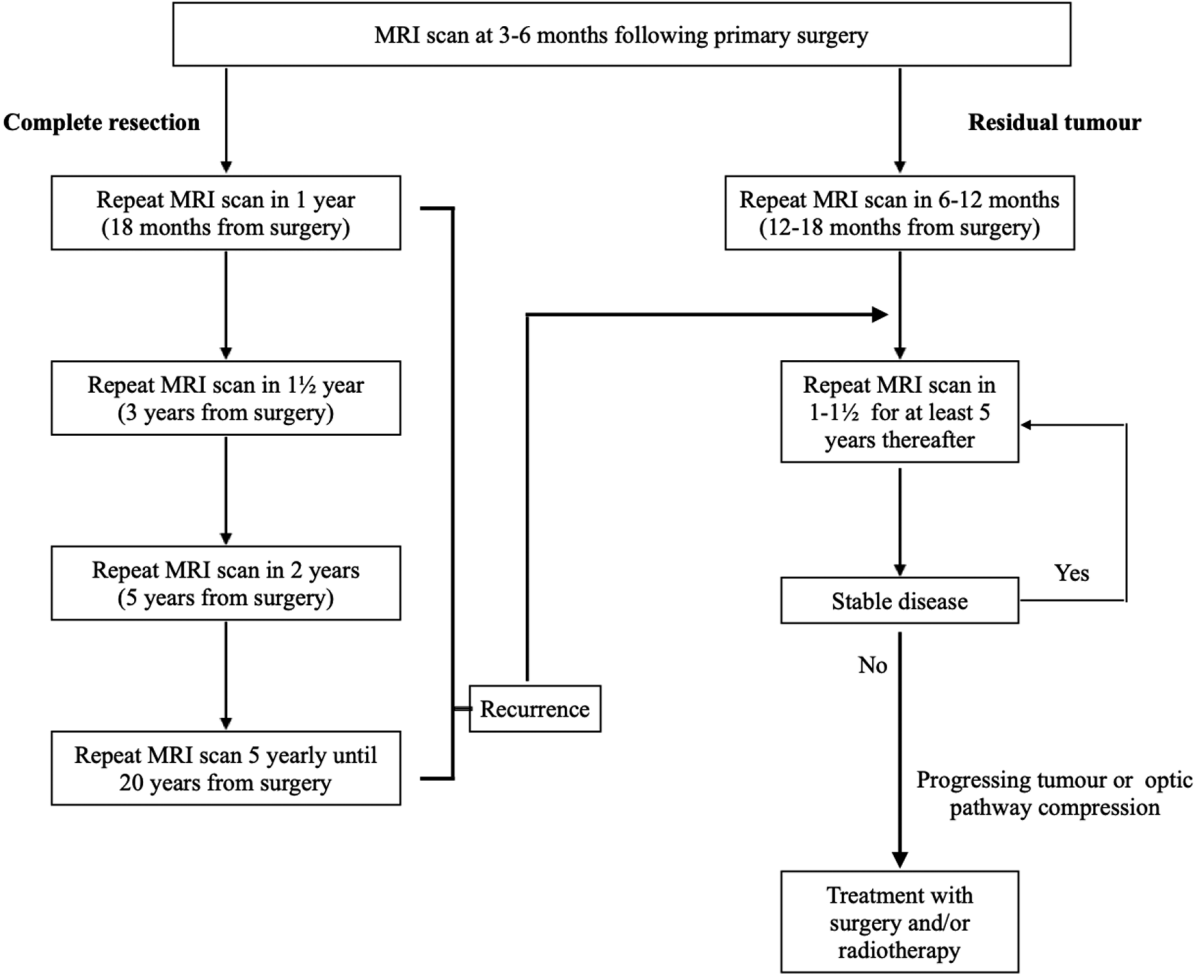
Neuroimaging follow-up strategy for patients who underwent primary surgery for non-functioning pituitary macroadenomas. MRI, magnetic resonance imaging.

This retrospective cohort study of 383 patients with NFPMs reports the following principal findings: (1) Residual disease on the first post-operative scan was a significant risk factor for NFPMs regrowth and associated with a higher risk of re-operation and radiotherapy; and (2) Ki-67 antigen expression in the resected pituitary adenoma was not a significant risk factor for re-operation, albeit higher in young patients and those that went on to receive radiotherapy. With these findings in mind, we propose a follow-up strategy that stratifies patients at “low risk” if there is no residual adenoma, with increasing scan intervals, or at “high risk” if there is a residual disease, with annual scans for at least five years and extended surveillance after that (Figure 4). In patients with growing residual disease, we consider re-operation if there is compression of the optic apparatus; and re-operation and/or radiotherapy in patients who are young enough such that future compression is anticipated.

### Comparison with other studies

Pituitary adenomas are re-termed PitNET in the 5th Edition of the WHO Classification of Endocrine and Neuroendocrine Tumors (1). Non-functioning subtype is shown to recur following surgery more frequently than other types of pituitary adenomas (11). The lack of hormone hypersecretion-related syndromes impedes early diagnosis and leads to delayed presentation with large tumours. NFPMs commonly manifest by invading nearby critical structures and compressing the optic pathway. Total resection of NFPMs is challenging, particularly while operating on invasive tumours extending beyond the sella turcica. Therefore, long-term follow-up with regular clinical and radiological assessment is necessary to detect disease progression and manage with further surgery with and without radiotherapy when appropriate.

Several studies attempted to establish robust clinicopathological and radiological factors to predict NFPMs recurrence after surgery. However, there is no consensus about these variables (16). Consequently, defining a clear neuroimaging time frame after surgery when following patients with NFPMs is still lacking. Young age, cavernous sinus invasion and partial resection of NFPMs are independent predictive factors of recurrent growth detected in this study. Similar findings were demonstrated by others (17–19).

On the contrary, Yeo et al. (20) and Batista et al. (21) studies have not related age to tumour recurrence, whereas Dubois et al. (22) reported that remanent tumours didn't influence recurrence in non-functional gonadotroph adenoma. In general, younger patients are more likely to have closer surveillance and radical treatment when developing NFPMs progression following surgery, while elderly patients have a more conservative approach. Patients with residual disease were at higher risk of further surgical intervention for regrowing tumours than those who underwent total resection. In addition, as demonstrated in this study, those with remanent tumours required surgical resection earlier than residual-free patients.

Transsphenoidal surgery is used in resecting most pituitary tumours. To date, two operative visualisation techniques exist: microscopic and endoscopic. The operating microscope was introduced in the late 1960s (23) and was the gold standard for resecting pituitary tumours over the next 30 years. Endoscopic transsphenoidal surgery is an alternative route introduced in the 1990s (24) with two approaches, endonasal and sublabial. There has been a growing trend of resecting pituitary adenomas using the endoscope over the last two decades during primary surgery and resecting progressing residual disease, given its enhanced visualisation, particularly into the cavernous sinus and suprasellar region, offering the potential for a greater degree of tumour removal than the microscopic approach. To this end, many studies have demonstrated a higher rate of complete surgical removal of pituitary adenomas, the most crucial prognostic outcome, using endoscopic surgery than microscopic modality (25–27). In this cohort, early patients predominantly underwent microscopic pituitary surgery, as two of the neurosurgeons operated using this approach. Endoscopic surgery was introduced in our centre in 2012, and all the current neurosurgeons are using this technique, with a corresponding improvement in resection rates demonstrated in this cohort. Transcranial surgery is rarely indicated but may have a role for patients with large pituitary adenomas and substantial temporal or anterior cranial fossa extension (28). Combined transsphenoidal and transcranial approaches can also be used in some circumstances.

Computer-guided neuronavigation and intraoperative imaging have also been increasingly deployed in pituitary surgery to achieve the best surgical outcome. Conducting transsphenoidal surgery with intraoperative MRI remains contentious due to increased cost and length of surgery, but it may more readily demonstrate the extent of tumour resection and increase the rate of complete resection, particularly in large tumours and in cases of recurrent adenomas (29, 30). In addition, MRI-guided resection is shown to have a low recurrence rate (29). Other intraoperative imaging using CT or ultrasound guidance have also been used, with the latter offering the advantage of being more readily integrated into the operative workflow and avoiding ionising radiation (31).

Definite molecular risk factors for NFPMs recurrence still need to be well established. The ki-67 antigen is considered a marker of tumour proliferation and invasiveness in pituitary tumours (32). However, its prognostic value in predicting recurrence and regrowth is debatable (32–34). The World Health Organization's classification of pituitary adenomas and recommendation for the behaviour of invasive tumours propose a cut-off of Ki-67 proliferation index >3% (35) as a factor of aggressive pituitary adenomas. In this study, recurrent NFPMs had a higher Ki-67 (>3%) than non-recurrent tumours, albeit not significantly raised. Furthermore, the proliferation index was higher in younger patients and those that went on to receive radiotherapy but not in those with cavernous and sphenoid sinus invasion compared to patients with macroadenoma confined to the sellar space. The relationship between Ki-67 antigen and age, pituitary adenoma recurrence and aggressive tumour behaviour has been controversial. Kyung-II Paek et al. (34) and Mastronardi et al. (36) couldn't correlate between Ki-67 and age in patients with pituitary adenomas. In contrast, other studies recorded higher expression in young patients (22, 37, 38), which is concordant with our study*.* Hongegger et al. (39) reported an independent correlation between Ki67 and invasiveness in NFPAs, while Chiloiro et al. (40) concluded that Ki-65 expression of more than 1.5% is associated with a greater risk of cavernous and sphenoid sinus invasion, which is in contrast to our findings, but their study included a cohort of functioning and non-functioning pituitary adenomas which can bias their data. Trouillas et al. (33), in their multicentric case-control study, developed the five-tiered prognostic clinicopathological classification of pituitary adenomas. The classification is based on pituitary MRI for cavernous or sphenoid sinus invasion and tumour size, immunocytochemistry analysis and proliferation markers (Ki-67, mitoses, and P 53). Tumours were classified according to size (microadenoma, macroadenoma and giant pituitary adenomas), type (Prolactin, Growth Hormone, FSH/LH, ACTH and TSH) and grade (grade 1a: non-invasive, 1b: non-invasive and proliferative, 2a: invasive, 2b: invasive and proliferative, and 3: metastatic). Sahakian and Raverot et al. (1, 21) assessed and validated the impact of this classification on tumour progression in invasive and proliferative PitNETs.

Postoperative pituitary radiotherapy provides excellent control of non-functioning adenoma regrowth with a potential reduction in tumour volume in a small proportion of cases. However, its routine use has been controversial and limited as adjuvant therapy for progressive and invasive tumours when surgery is unfeasible. In this study, the probability of tumour progression-free survival after radiotherapy was comparable to the literature (8). Age and cavernous sinus involvement influenced the need for radiotherapy for our patients. Chang et al. (41) also reported that those with adenomas invading the cavernous sinus needed radiotherapy more frequently than those without (41). The potential risk of hypopituitarism, injury to nearby radiosensitive structures, late development of secondary brain tumours and cardiovascular accidents should be considered when radiotherapy is used. In our centre, all patients are discussed in the pituitary multidisciplinary meeting to assess the need for surgery with careful consideration of using radiotherapy. We currently deploy proton beam therapy to treat children and young adults up to their 25th birthday to reduce the late effects of radiation.

Surgery is very effective in relieving compressive visual symptoms, with 90% of patients having normal or improved symptoms after presenting with a visual deficit with an extremely low incidence of neuro-ophthalmic deterioration, as demonstrated in this series. This is keeping with the literature (42–44). Radiation-induced optic neuropathy is a rare complication of pituitary adenoma irradiation (8, 45); none were reported in this cohort.

## Strengths and limitations

This study was based on a retrospective review of medical records. Therefore, it is liable to retrospective study weaknesses, including data loss and selection bias. In particular, a large number of patients' immediate post-operative imaging were reported at the time as demonstrating residual sellar tissue of uncertain significance. Our post-hoc decision to group residual sellar tissue of uncertain significance with complete resection will have impacted the interpretation of results. In addition, the study included gonadotropin expressing and pleurihormonal adenomas and not the whole spectrum of NFPMs. In this cohort, P53 was not assessed routinely in all patients; therefore, the five-tiered prognostic classification of pituitary adenomas can not be applied in this study. Nevertheless, this study included a large cohort of patients with NFPMs with a long follow-up duration with generalisable findings. It demonstrates the natural course of NFPMs following surgery and radiotherapy, as well as establishing potential clinical and radiological factors to develop neuroimaging follow-up strategy.

## Conclusions

We demonstrated that NFPMs recurrence and regrowth are common following surgery. Age, cavernous sinus invasion, and corresponding partial resection are significant risk factors for disease progression and might be helpful variables in proposing long-term radiological surveillance. Radiotherapy is very effective in managing regrowth of tumour remnant, but the risk of complications should be considered. Based on our study findings, we propose a follow-up strategy that stratifies patients at “low risk” if there is no residual adenoma, with increasing scan intervals, or at “high risk” if there is a residual disease, with annual scans for at least five years and possible extended lifelong surveillance (Figure 4).

## Data Availability

The original contributions presented in the study are included in the article/Supplementary Materials, further inquiries can be directed to the corresponding author/s.
